# Molecular studies of phages-*Klebsiella pneumoniae* in mucoid environment: innovative use of mucolytic agents prior to the administration of lytic phages

**DOI:** 10.3389/fmicb.2023.1286046

**Published:** 2023-10-11

**Authors:** Olga Pacios, Lucía Blasco, Concha Ortiz Cartagena, Inés Bleriot, Laura Fernández-García, María López, Antonio Barrio-Pujante, Felipe Fernández Cuenca, Belén Aracil, Jesús Oteo-Iglesias, María Tomás

**Affiliations:** ^1^Grupo de Microbiología Traslacional y Multidisciplinar (MicroTM)-Servicio de Microbiología Instituto de Investigación Biomédica A Coruña (INIBIC), Hospital A Coruña (CHUAC), Universidad de A Coruña (UDC), A Coruña, Spain; ^2^Grupo de Estudio de los Mecanismos de Resistencia Antimicrobiana (GEMARA) formando parte de la Sociedad Española de Enfermedades Infecciosas y Microbiología Clínica (SEIMC), Madrid, Spain; ^3^Unidad Clínica de Enfermedades Infecciosas y Microbiología Clínica, Hospital Universitario Virgen Macarena, Instituto de Biomedicina de Sevilla (Hospital Universitario Virgen Macarena/CSIC/Universidad de Sevilla), Sevilla, Spain; ^4^MePRAM, Proyecto de Medicina de Precisión contra las resistencias Antimicrobianas, Madrid, Spain; ^5^Laboratorio de Referencia e Investigación de Resistencias a Antibióticos e Infecciones Sanitarias, Centro Nacional de Microbiología, Instituto de Salud Carlos III, Madrid, Spain; ^6^CIBER de Enfermedades Infecciosas (CIBERINFEC), Instituto de Salud Carlos III, Madrid, Spain

**Keywords:** *Klebsiella pneumoniae*, lytic bacteriophages, phage resistance, co-evolution, mucin, n-acetyl cysteine

## Abstract

Mucins are important glycoproteins that form a protective layer throughout the gastrointestinal and respiratory tracts. There is scientific evidence of increase in phage-resistance in the presence of mucin for some bacterial pathogens. Manipulation in mucin composition may ultimately influence the effectiveness of phage therapy. In this work, two clinical strains of *K. pneumoniae* (K3574 and K3325), were exposed to the lytic bacteriophage vB_KpnS-VAC35 in the presence and absence of mucin on a long-term co-evolution assay, in an attempt to mimic *in vitro* the exposure to mucins that bacteria and their phages face *in vivo*. Enumerations of the bacterial and phage counts at regular time intervals were conducted, and extraction of the genomic DNA of co-evolved bacteria to the phage, the mucin and both was performed. We determined the frequency of phage-resistant mutants in the presence and absence of mucin and including a mucolytic agent (N-acetyl L-cysteine, NAC), and sequenced them using Nanopore. We phenotypically demonstrated that the presence of mucin induces the emergence of bacterial resistance against lytic phages, effectively decreased in the presence of NAC. In addition, the genomic analysis revealed some of the genes relevant to the development of phage resistance in long-term co-evolution, with a special focus on the mucoid environment. Genes involved in the metabolism of carbohydrates were mutated in the presence of mucin. In conclusion, the use of mucolytic agents prior to the administration of lytic phages could be an interesting therapeutic option when addressing *K. pneumoniae* infections in environments where mucin is overproduced.

## 1. Introduction

*Klebsiella pneumoniae* is a Gram-negative opportunistic pathogen that causes urinary tract, wound and soft tissue infections, pneumonia, and even life-threatening sepsis (Kamruzzaman and Iredell, 2019; Mackow et al., 2019). Moreover, the recent increase of carbapenemase-producing strains of *K. pneumoniae* worldwide, together with its ability to grow in biofilm and to acquire plasmids conferring antibiotic (multi)resistance, underlie the importance of developing innovative and effective strategies against *K. pneumoniae* infections (Majkowska-Skrobek et al., 2021; Pacios et al., 2021).

In this context, the use of bacteriophages (or phages), viruses that specifically target bacteria in a highly effective and safe manner, is being evaluated as a therapeutic approach against bacterial infections, especially antibiotic-resistant ones (Pacios et al., 2020; Majkowska-Skrobek et al., 2021). Nevertheless, just as it happens with antibiotics, the emergence of phage-resistant mutants is a major hurdle to the establishment of phage therapy (Majkowska-Skrobek et al., 2021; Uyttebroek et al., 2022). Indeed, to counter phage infection, bacteria display several defence mechanisms: mutation of the receptor recognized by a particular phage to inhibit adsorption (surface mutation) (Denes et al., 2015), induction of programmed cell death, known as abortive infection (Abi) (Lopatina et al., 2020), translation of nucleases that specifically degrade the phage DNA [CRISPR-Cas, restriction-modification… (Castillo et al., 2020; Ambroa et al., 2021)], etc. Despite the inconvenience of resistant bacteria against phages, their compassionate use in clinics has been approved in many countries and has already saved many life-threatening infections in patients (Schooley et al., 2017; Dedrick et al., 2019; Law et al., 2019).

Cystic Fibrosis (CF), an autosomal recessive genetic disorder that produces mutations in the cystic fibrosis transmembrane conductance regulator (CFTR) protein, is characterized by an overproduction of viscous mucins, since lack of CFTR function reduces airway mucus fluidity and influences hydration and mucin viscosity in the airways (Riquelme et al., 2018). This allows the trapping of inhaled bacteria in the lungs and explains why CF patients often become colonized by pathogens from an early age, which can lead to chronic infections (Pletzer et al., 2017). Even if a few typical bacteria are traditionally involved in CF lung infections, such as *Staphylococcus aureus* and *Pseudomonas aeruginosa*, CF patients are susceptible to infection by other opportunistic pathogens, including *K. pneumoniae* (Leão et al., 2011; Delfino et al., 2015). Especially relevant are the hypervirulent *K. pneumoniae* strains, in which hypermucoviscosity plays a key role in their pathogenesis and immune evasion, as shown in human and animal serum survival assays (Xu et al., 2021).

To improve therapeutic outcomes in phage therapy, the arising of phage-resistant bacteria in the complex *in vivo* context needs to be exploited. Furthermore, not many studies address the efficiency of phage in long-term evolutionary experiments, nor look at phage co-evolution during phage treatments, as reviewed by Moulton-Brown and Friman (2018).

One of the main components of the gastrointestinal and respiratory tracts are mucins. Mucins are high-molecular-weight proteins that are glycosylated and can be transmembrane (forming a protective “brush” border on the epithelium) or gel-forming (providing hydration and protection from shear stress) (Hansson, 2019; Paone and Cani, 2020). They protect the intestinal mucosa from physical contact with commensal bacteria, as well as from invasion of intruders and pathogens (Carroll-Portillo and Lin, 2021). Changes in mucin expression are relevant in inflammatory and neoplastic disorders of the gastrointestinal tract, being important in the etiology of some infectious diseases, such as *Helicobacter pylori* gastritis (Jass and Walsh, 2001).

In the present work, we have used two bacteriemia-causing clinical isolates of *K. pneumoniae*, named K3574 and K3325, and exposed them to the lytic bacteriophage vB_KpnS-VAC35 [previously characterized by our group (Bleriot et al., 2023)] in the presence and absence of mucin on a long-term co-evolution assay, intending to study the phage resistance in a mucoid environment. We determined the relationship between mucin and the difficulties in applying phage therapy, and we included the mucolytic agent N-acetyl cysteine (NAC) to improve the use of phages by avoiding the emergence of resistance.

## 2. Materials and methods

### 2.1. Bacterial strains and growth conditions

*K. pneumoniae* clinical strains K3574 and K3325 were isolated from patient’s blood and stored at the National Centre for Microbiology (Carlos III Health Institute, Spain), the former being the one of choice for the phenotypic characterization of the lytic bacteriophage vB_KpnS-VAC35 (Bleriot et al., 2023). All the bacterial strains were cultivated using Luria-Bertani broth (LB, 1% tryptone, 0.5% yeast extract and 0.5% NaCl). When required, purified mucin from porcine stomach (SigmaAldrich^®^), previously diluted in distilled water and autoclave-sterilized, was added at a final concentration of 1 mg/mL. NAC was also purchased from SigmaAldrich^®^, diluted with nuclease-free water, filter-sterilized and added (when corresponded) to a final concentration of 10 mM. The absence of interaction between NAC and the bacteriophage was confirmed with a growth curve of the clinical isolate K3574 (Supplementary Figure S1).

### 2.2. Establishment of the infectivity of the phage

#### 2.2.1. Efficiency of plating

The EOP assay was done as previously described (Kutter, 2009), calculated as the ratio between the phage titre (plaque forming units, PFU/mL) in the test strain and the titre in the isolation host (*K. pneumoniae* K3574). For both assays, TA-soft medium (1% tryptone, 0.5% NaCl and 0.4% agar) was used to make plates by the top-agar method (Abedon and Yin, 2009). Strains exhibiting susceptibility to phage infection in the spot test performed by Bleriot et al. in a previous work from our group were selected for the EOP assay (Bleriot et al., 2023).

#### 2.2.2. Infection curves

To assess the lytic capacity of vB_KpnS-VAC35, infection curves at different multiplicities of infection (MOI) were performed. Overnight cultures of the clinical isolates of *K. pneumoniae* K3574 and K3325 were diluted 1:100 in LB broth and then incubated at 37°C at 180 rpm until an early exponential phase (OD_600nm_ = 0.3–0.4) was reached. Then, vB_KpnS-VAC35 was added to the cultures at MOI of 0.1 and 1, and OD_600nm_ was measured during 6 h at 1 h intervals.

### 2.3. Co-evolution between vB_KpnS-VAC35 and *Klebsiella pneumoniae* strains K3574 and K3325

The bacterial strains were incubated in 20 mL LB-containing flasks at 37°C and 180 rpm for 6 (K3325) or 15 days (K3574), re-inoculated daily into fresh LB medium (1:100 dilution). The flasks were infected with vB_KpnS-VAC35 at a MOI = 1 in the presence and absence of porcine mucin at a final concentration of 1 mg/mL, and a non-infected control of the bacterial isolate growing in presence of 1 mg/mL mucin was included. The infections with the phage were performed at OD ≈ of 0.4. From this moment and every 24 h, each condition was 1:100 diluted in fresh LB medium, containing 1 mg/mL mucin when required, and enumeration of colony forming units (CFU) and PFU was performed. For the CFU enumeration, 1 mL aliquots of bacterial cultures were serially diluted in the saline buffer then platted on LB-agar plates (100 μL) and incubated overnight. For the PFU assessment, 1 mL aliquots were centrifuged 5 min at maximum speed (14,000 rpm) for the collection of phage particles in the supernatant. Serial dilutions of these PFU were performed in SM buffer (100 mM NaCl, 10 mM MgSO4, 20 mM Tris-HCl, pH 7.5), then 10 μL of the pertinent dilutions were plated by the double-layer method and enumerated after overnight incubation (Abedon and Yin, 2009). Two flasks per condition were considered as biological duplicates.

### 2.4. Assessment of phage resistance

#### 2.4.1. Spot test

The spot test assay was undertaken as described by Raya and H’bert (2009). We used vB_KpnS-VAC35 WT, vB_KpnS-VAC35_ad15 and vB_KpnS-VAC35_ad15_m phages, that is prior to co-evolution, and adapted to K3574 during 15 days in the absence and presence of mucin, respectively.

#### 2.4.2. Calculation of the frequency of phage-resistant mutants

The frequency of resistant mutants was calculated as previously described by Lopes et al., (2018). Overnight cultures of the strains K3574 and K3325 at the different conditions evaluated were diluted 1:100 in LB and grown to an OD_600nm_ of 0.7. An aliquot of 1 mL of the culture containing 10^8^ CFU/mL was serially diluted, and the corresponding dilutions were mixed with 100 μL of vB_KpnS-VAC35 at 10^9^ PFU/mL, then plated by the double-layer method in TA medium. The plates were incubated at 37°C for 24 h, then the colonies of resistant mutants were enumerated. The mutation rate was calculated by dividing the number of resistant bacteria (growing in the presence of the phage) by the total number of bacteria plated in conventional LB-agar (100 μL).

### 2.5. Genomic DNA extraction and whole-genome sequencing

The DNeasy Blood & Tissue Kit (Qiagen^®^) was used for extracting the genomic DNA of bacterial cultures co-evolved 15 dpi with the vB_KpnS-VAC35 alone (K3574_ad15_P), in the presence of mucin (K3574_ad15_P + M) and exposed 15 days to mucin (K3574_ad15_M), following the manufacturer’s instructions. Samples were quantified with a Qubit 3.0 fluorometer using a Qubit dsDNA HS Assay Kit and with a Nanodrop spectrophotometer to evaluate the DNA purity.

The Oxford Nanopore MinION MK1C platform with the Rapid Barcoding Kit (SQK-RBK004) were employed to obtain the long reads. A Nanopore sequencing library was made, and the pooled samples were loaded onto the FLO-MIN106 R9 (v.9.4.1) flowcell, following the manufacturer’s instructions. A 48 h run was conducted without real time basecalling since this step was performed afterwards (see below).

### 2.6. Bioinformatic analysis

**Figure 1:**
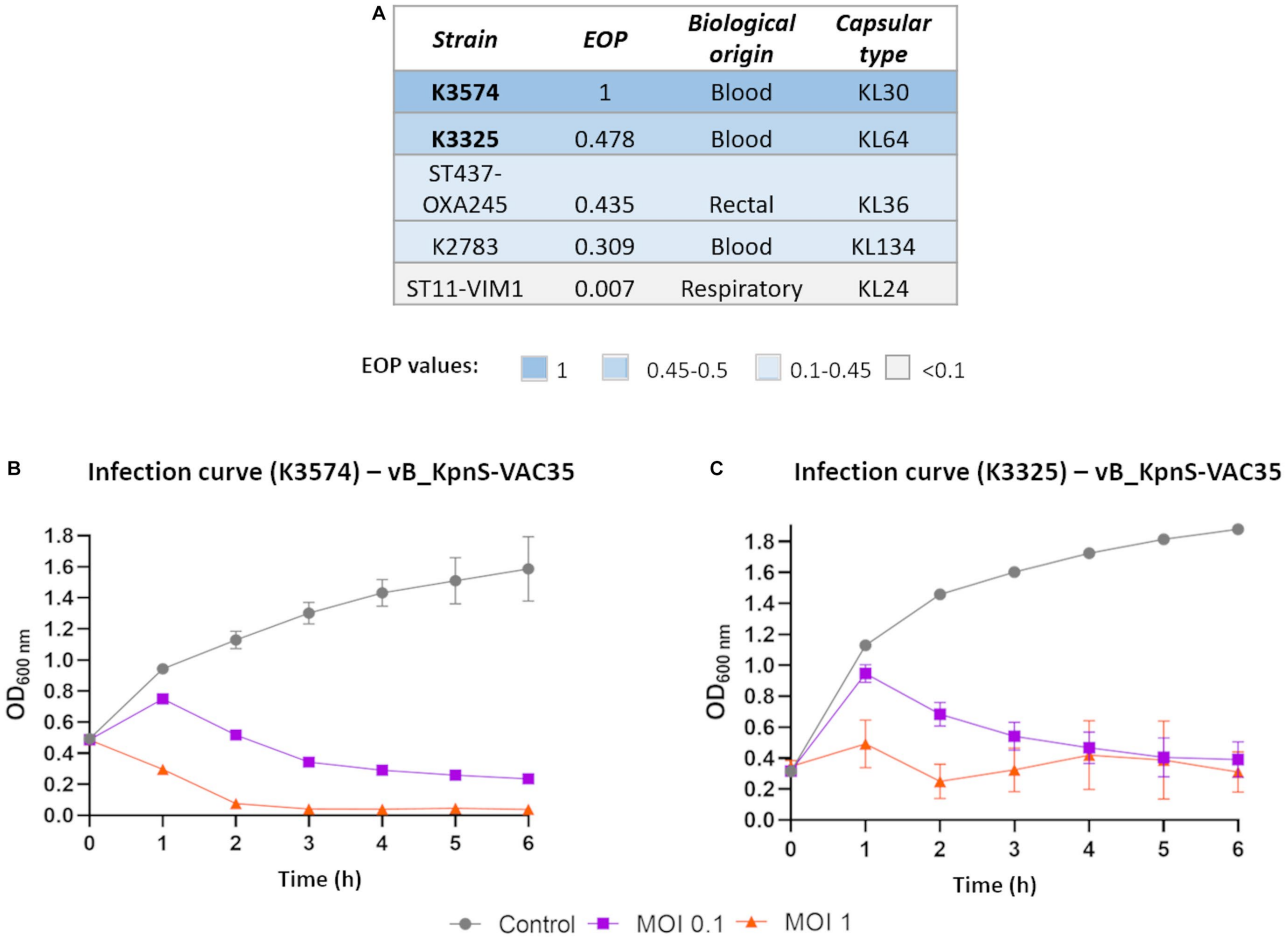
**(A)** EOP of vB_KpnS-VAC35 on the clinical isolates of *K. pneumoniae* that exhibited a positive spot test. **(B)** and **(C)** Infection curves of clinical isolates K3574 and K3325, respectively, by the lytic phage vB_KpnS-VAC35, prior to co-evolution, at MOI 0.1 and 1.

Basecalling was performed using GUPPY (Version 5.0.7 Super-accuracy model (SUP)) to generate fastQ sequencing reads from electrical data (the fast5 files generated by MinION). Quality control of the generated fastQ reads was performed using the FastQC^1^ and NanoPlot^2^ tools. The reads were then further subsampled according to their barcodes and *de novo* assembled using Unicycler (v1.0+). Draft assemblies were corrected by iterative rounds of polishing with the error correction software Racon. Subsequently, the Bandage tool was used in order to assess the quality of assemblies quickly and visually. Annotations were performed using Prokka (Seemann, 2014), and insertions, deletions and other SNPs were called using the structural variant caller Snippy (v1.0.11). The presence and absence of intact genetic sequences were analyzed using Roary and Orthovenn2.^3^ OrthoVenn2 was used to compare the proteins of the four complete genomes using the files generated by Prokka analysis. Fasta files obtained after annotation were surveilled for indels using the blastn and blastp tools from the NCBI and compared to the reference genome (for K3574_WT, BioSample code SAMEA3649560 included in the European BioProject PRJEB10018). The workflow taken from Nanopore sequencing to the genomic analysis is summarized in Figure 1A.

## 3. Results

### 3.1. Infectivity of phage vB_KpnS-VAC35

**Figure 2:**
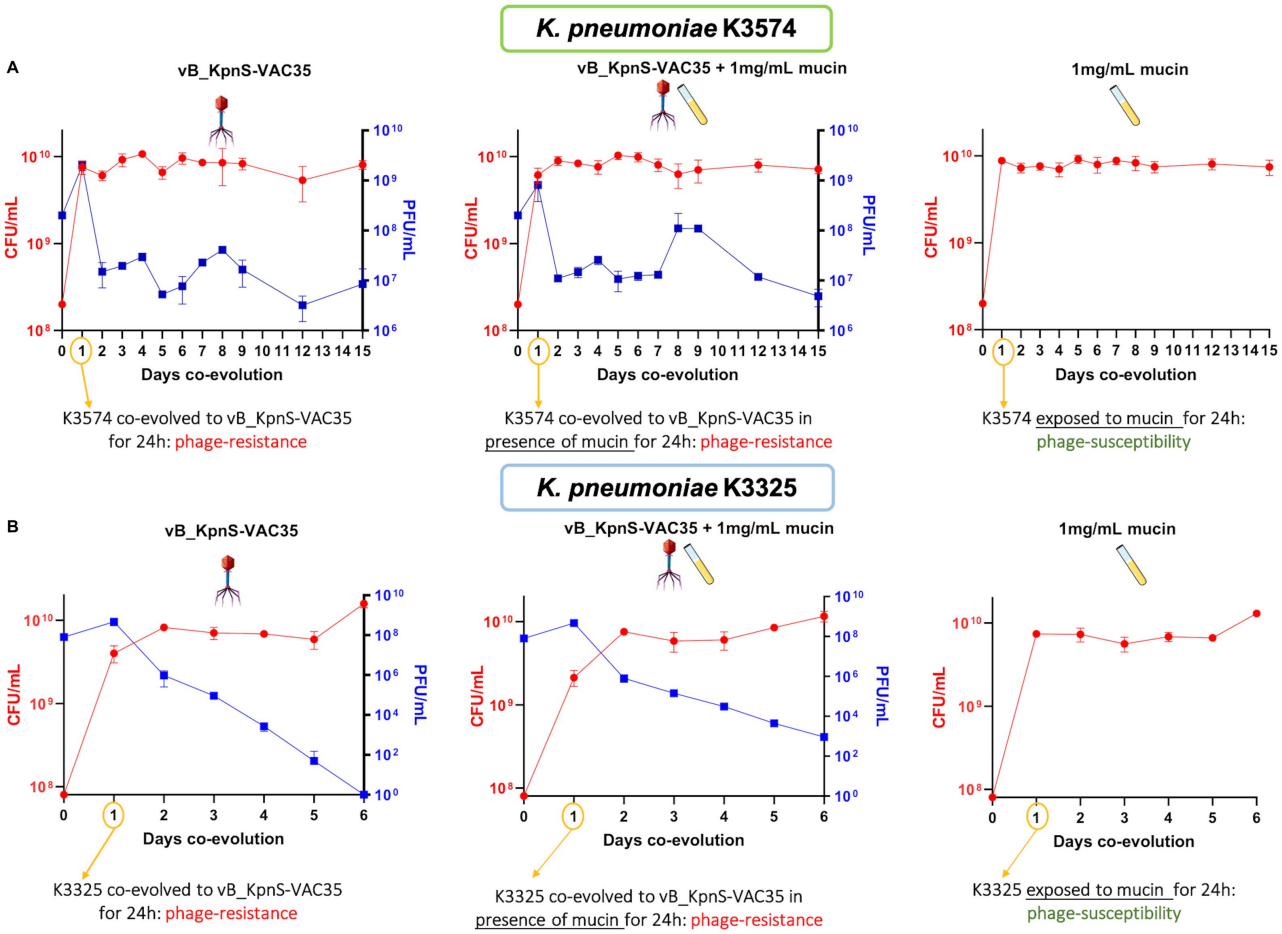
Titration of the colony forming units (CFU, left *Y*-axis) and plaque forming units (PFU, right *Y*-axis) per mL during the co-evolution experiments between clinical isolates K3574 **(A)** and K3325 **(B)** and the lytic bacteriophage vB_KpnS-VAC35, in the absence and presence of mucin (1 mg/mL).

The two strains exhibiting the highest EOP values (K3574 and K3325) were chosen for further assays (Figure 2A). Optical density growth curves showed good lytic activity of vB_KpnS-VAC35 in these strains at MOI of 0.1 (purple line) and 1 (orange line) (Figures 2B,C, respectively). The isolate K3325 was less susceptible to the lytic phage vB_KpnS-VAC35 than the isolation host, K3574.

### 3.2. Co-evolution of K3574 and K3325 with the phage vB_KpnS-VAC35 in a mucoid environment

**Figure 3:**
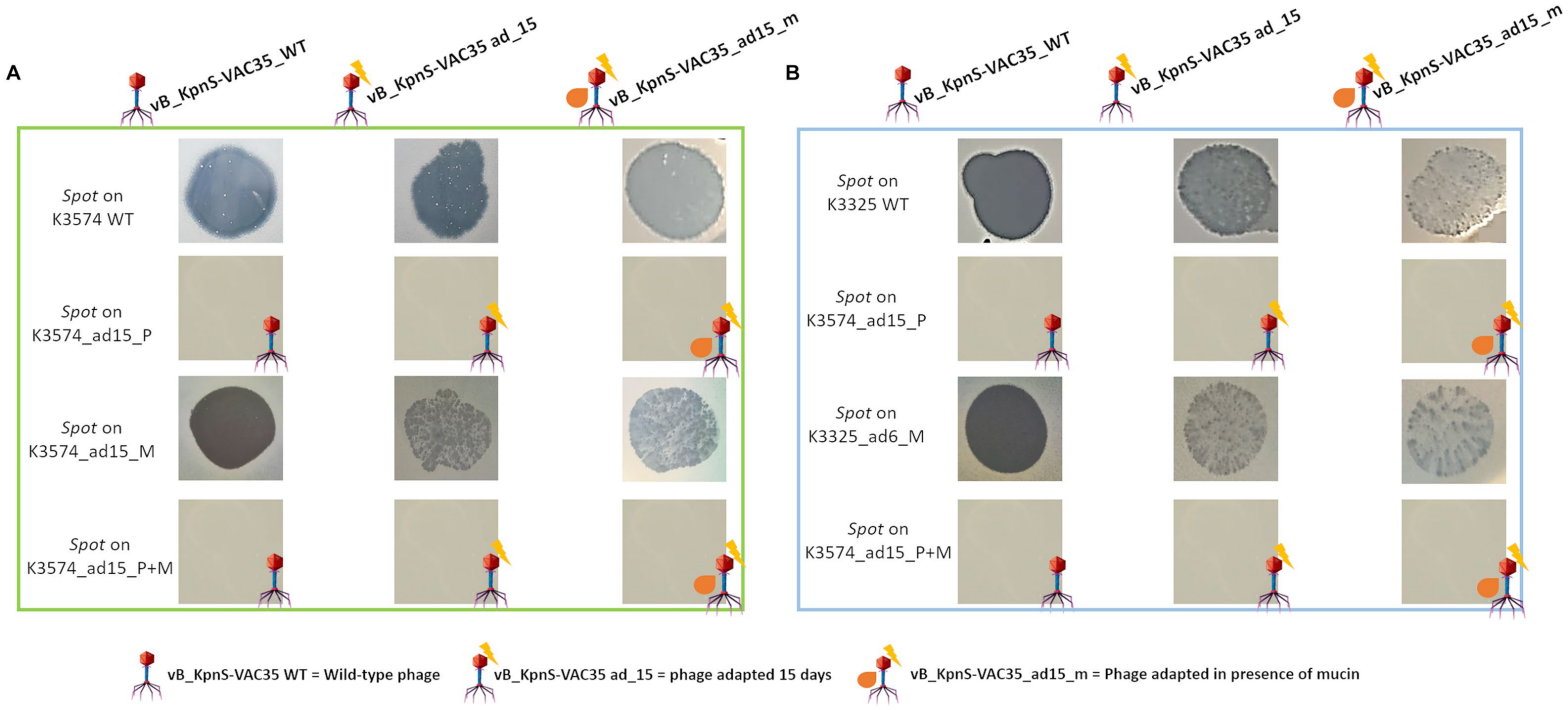
Spot tests of phages prior to co-evolution (vB_KpnS-VAC35 WT), co-evolved to K3574 in the absence and in the presence of mucin (vB_KpnS-VAC35_ad15 and vB_KpnS-VAC35_ad15_m, respectively). **(A)** Spot test using the clinical isolate *K. pneumoniae* K3574 WT, adapted 15 days to the phage, to mucin and to both. **(B)** Spot test using the clinical isolate *K. pneumoniae* K3325 WT, adapted 6 days to the phage, to mucin and to both.

For the co-evolution experiment, the initial bacterial inoculum was 2 × 10^8^ CFU/mL for K3574 and 10^8^ CFU/mL for K3325 (Figure 3). As we infected both cultures in the exponential growth phase at a MOI = 1, the initial phage concentration was 2 × 10^8^ PFU/mL and 10^8^ PFU/mL, respectively (Figure 3). The CFU counts remained stable at around 10^10^ CFU/mL for both strains at every condition tested, whereas the PFUs fluctuated slightly more: in what concerns the isolate K3574 co-adapted to the phage, PFU counts ranged from 10^9^ PFU/mL at 1 day post-infection (dpi) to 10^7^ PFU/mL at 15 dpi, whereas in the presence of mucin these counts reached 10^8^ PFU/mL at 9 dpi, then slightly decreased till 10^7^ PFU/mL at 15 dpi (Figure 3A), which corresponds to the 1:100 dilution performed every day along the experiment. Regarding the isolate K3325, co-evolution lasted 6 days as the PFU numbers dropped to 0 in the absence of mucin (Figure 3B). Nonetheless, in the presence of this compound, 10^3^ PFU/mL of vB_KpnS-VAC35 were assessed at 6 dpi.

### 3.3. Assessment of phage-resistance

#### 3.3.1. Spot test

**Figure 4:**
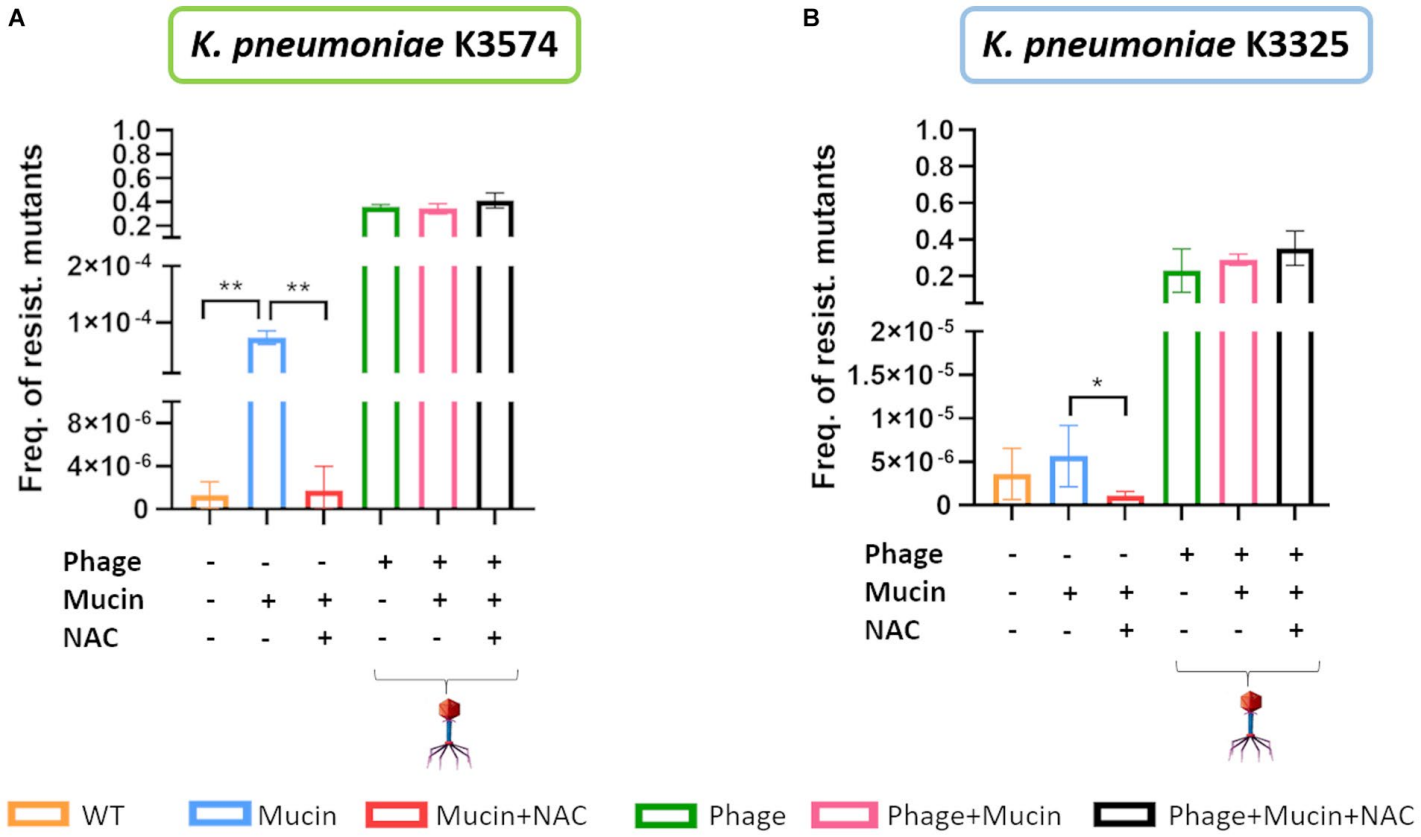
Frequency of occurrence of resistant mutants for *K. pneumoniae* K3574 **(A)** and *K. pneumoniae* K3325 **(B)**. The statistical analysis (*t*-test) was performed with GraphPad Prism v.9. **value of *p* <0.001 and *value of *p* 0.042. Absence of asterisk corresponds with no statistical significance.

In order to evaluate how the presence of mucin will affect the bacterial susceptibility to the adapted phages a spot test of *K. pneumoniae* K3574 and K3325 prior to the co-evolution (named as “WT” in Figure 4), but also co-evolved in the presence of mucin, the phage, and both during 15 and 6 days, respectively, was conducted. The only conditions in which phage-susceptibility was kept was for WT and mucin-adapted cells (K3574_ad15_m and K3325_ad6_m), in which no phage-infection was established (Figure 4). However, we observed differences when comparing the infection established by the non-adapted phage (vB_KpnS-VAC35_WT) and the adapted ones (vB_KpnS-VAC35_ad15 and vB_KpnS-VAC35_ad15_m), which were isolated after 15 days of co-evolution with K3574 and produced more turbid spots (Figure 4, middle and right columns). The presence of more colonies growing inside the lytic halos of vB_KpnS-VAC35_ad15_m compared to the infection established by vB_KpnS-VAC35_ad15 suggested that mucin either affected the phage-infection capacity, or it enhanced the bacterial defence to the phage [as it has already been documented in the literature for different microorganisms (Green et al., 2021; de Freitas Almeida et al., 2022)].

#### 3.3.2. Frequency of arising of resistant mutants in the presence of the mucolytic N-acetyl L-cysteine

**Figure 5:**
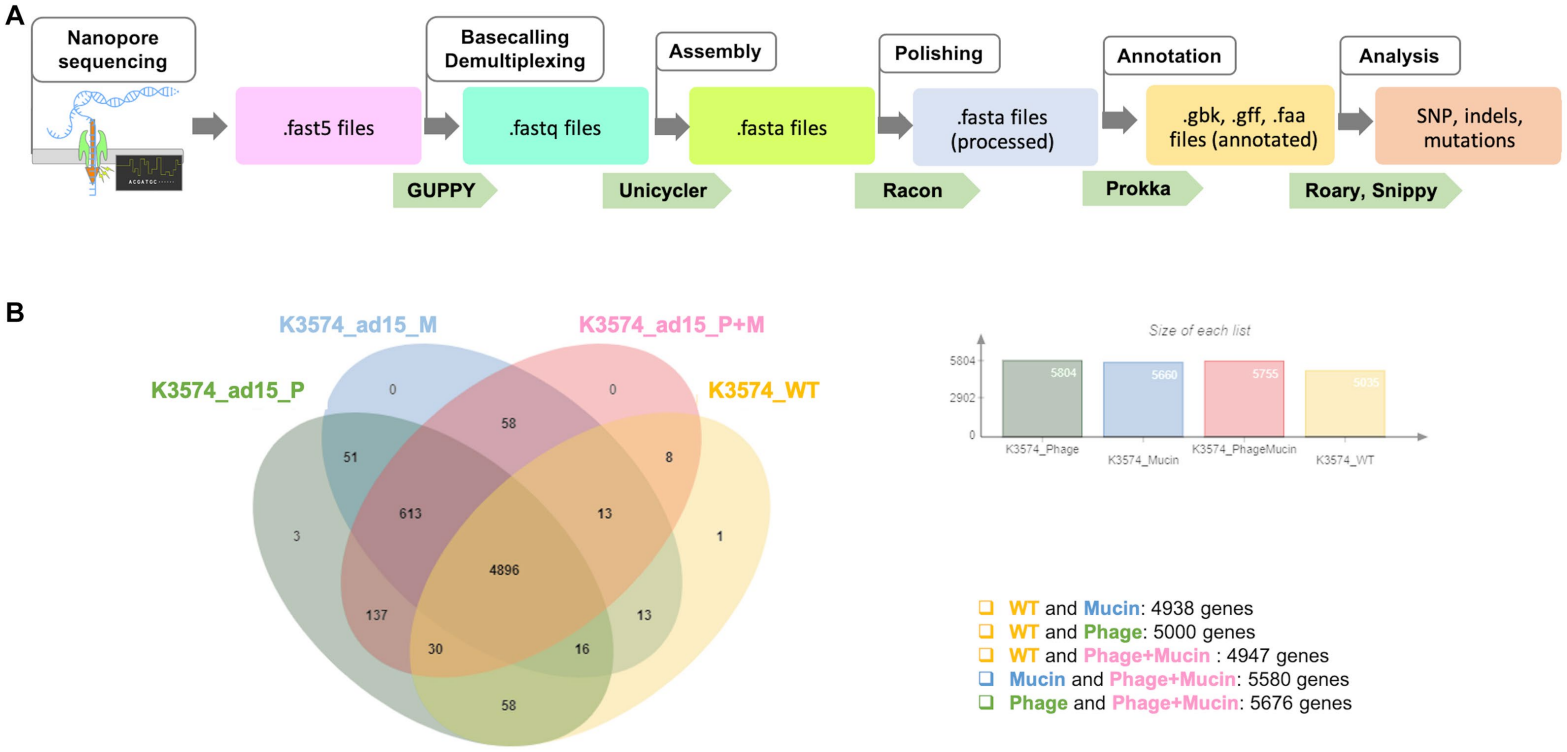
**(A)** Schematic representation of the workflow followed from the whole-genome sequencing with Nanopore till the analyse of the genomic sequences. **(B)** Venn diagram performed with OrthoVenn2 to visualize the overlapped and unique protein clusters in the four complete genomes analysed.

To quantitatively assess the effect that the presence of mucin had during bacteria and phage co-evolution, we determined the frequency of phage-resistant mutants to vB_KpnS-VAC35 in 6 different conditions, depicted in Figure 5: (i) WT (orange bar); (ii) exposed to mucin alone (blue bar); (iii) mucin and its inhibitor, NAC (red bar); (iv) phage alone (green bar); (v) phage and mucin (pink bar); and, finally, (vi) phage, mucin and the NAC inhibitor (black bar). Thus, the condition in which *K. pneumoniae* clinical isolates K3574 and K3325 were incubated in presence of mucin and NAC for 15 and 6 days, respectively, was included. Consistently with the infection curves of vB_KpnS-VAC35 in these two strains, we obtained a higher frequency for K3325 than K3574 (Figure 5, orange bar). In the case of phage-exposed bacteria, either in the presence of mucin alone, mucin and NAC, or in the absence of these compounds, no statistical difference was observed (Figure 5). Importantly, cells pre-exposed to mucin and NAC exhibited a statistically significant reduction in the phage-resistance frequency (Figure 5, red bar) compared to the cells exposed exclusively to mucin (Figure 5, blue bar, *p*-values <0.001).

### 3.4. Genomic analysis of *Klebsiella pneumoniae* strains before and after the co-evolution in the presence of mucin

Genomic analysis of the clinical strain K3574 adapted to mucin, to the phage alone or to both agents, were performed. We exclusively chose this strain, considered the “isolation host,” as previously said, because its co-evolution with the bacteriophage vB_KpnS-VAC35 lasted 15 days (instead of 6) and showed a higher susceptibility to the infection (Figure 2).

The reference genome of this strain (BioSample code SAMEA3649560, European BioProject PRJEB10018) possesses 5,635,279 bp (5,561 coding sequences) with a GC content of 57.1%, a sequence-type ST3647 and a capsular type KL30. We extracted the bacterial DNA at 15 dpi of this isolate co-evolved to the phage (K3574_ad15_P, deposited into GenBank under the BioSample code SAMN37179281 and the Genome Accession JAVLVI000000000), to mucin (K3574_ad15_M, deposited into GenBank under the BioSample code SAMN37179282 and the Genome Accession JAVLVH010000001) and to both (K3574_ad15_P + M, deposited into GenBank under the BioSample code SAMN37179283 and the Genome Accession JAVLVG000000000). These three sequences belong to the BioProject PRJNA1010120.

A Venn diagram was used to visualize the different and overlapping protein clusters displayed by the four complete genomes taken into consideration (Figure 1B). In total, 5,000 common protein clusters were found between the strain prior to co-evolution and bacteria co-evolved to the phage (K3574_ad15_P), whereas 4,938 were found between K3574_WT and the mucin-adapted cells (K3574_ad15_M), and 4,947 common protein clusters between K3574_WT and cells co-evolved to the phage in the presence of mucin (K3574_ad15_P + M). No specific protein clusters were found for the strain adapted only to mucin (K3574_ad15_M) and to phage and mucin together (K3574_ad15_P + M), while 3 unique protein clusters were found in the isolate co-evolved to the phage alone (K3574_ad15_P) (Figure 1B).

**Figure 6:**
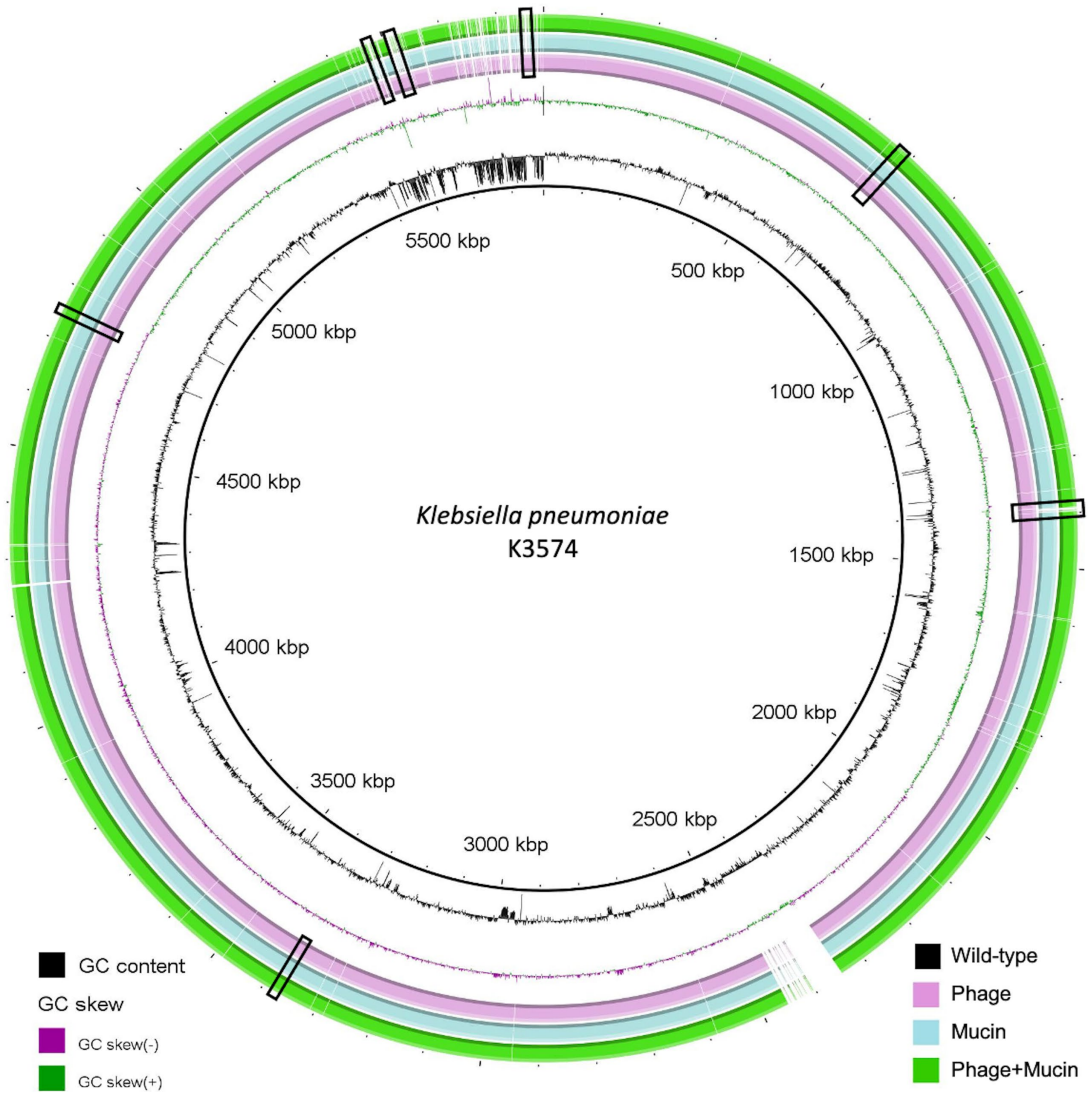
Comparative genomic analysis of *K. pneumoniae* K3574 co-evolved to phage alone (pink ring), mucin alone (blue ring) and both (green ring) constructed with the BLAST Ring Image Generator (BRIG). The sequence corresponding to K3574_WT is located on the innermost side (black ring). The double ring adjacent to the reference sequence represents the GC content (black) and the GC skew (dark purple and dark green). The white parts of the rings represent absent or divergent content and are squared in black.

Comparison of the genomes revealed important mutations (Figure 6 and Table 1). Among the genes in which nucleotide changes were found, we highlight several interesting ones grouped into different categories: concerning the bacterial defense mechanisms to phage infection, a tRNA-guanosine (18)-2’-O-methyltransferase carrying a nucleotide deletion in the position 362 was found in the case of K3574_ad15_P and K3574_ad15_P + M, whereas the non-infected isolate (K3574_ad15_M) had the intact locus compared to the K3574_WT. This change (c.-362G) leads to a frameshift mutation translated into two truncated versions of the methyltransferase. Furthermore, the antitoxin HigA displayed mutations in the phage-infected cultures (K3574_ad15_P and K3574_ad15_P + M) that led to two different truncated proteins, whereas the non-infected strain had fewer changes that led to a shorter, unique version. Furthermore, the autoinducer 2-binding protein LsrB presented the same nucleotide deletion in the strains that co-evolved to the phage, also corresponding to a frameshift mutation (therefore a truncated protein lacking 11 amino acids); this was absent in the isolate exposed only to mucin.

**Table 1:** Mutations found in the four complete genomes analyzed, grouped into different categories by function.

	K3574_ad15_P (co-evolved with the phage)	K3574_ad15_M (non-infected, exposed to mucin)	K3574_ad15_P + M (co-evolved with the phage in presence of mucin)
*Anti-phage bacterial defense*			
Anti-toxin HigA	c.T66-, c.T114-, c.A152- c.C199T, c.C201A. 2 truncated proteins	c.T66-, c.T105-, c.T106-. 1 protein FDNEPAPDTPEGDF>LIMNLRRTRRKG-I	c.T66-, c.T114-, c.A152- c.C199T, c.C201A. 2 truncated proteins
CRISPR-associated endonuclease/helicase Cas3	None	None	c.A2130-, c.A1236-, p.ALLAEVGVGTLDQLL>RYSLKL-VSVLSISF
tRNA (guanosine(18)-2’-O)-methyltransferase	c.A362-	None	c.A362-
*Quorum sensing*			
Autoinducer 2-binding protein LsrB	c.G341-, truncated protein (11 aa less)	None	c.G341-, truncated protein (11 aa less)
*PG synthesis*			
Murein DD-endopeptidase MepS/Murein LD-carboxypeptidase	Truncated version (lack 28 aas)	None	Truncated version (lack 28 aas)
FtsI Peptidoglycan D-transpeptidase	c.A1495- p.W501V	None	c.A1495- p.W501V
*LPS biosynthesis*			
undecaprenyl phosphate-alpha-4-amino-4-deoxy-L-arabinose arabinosyl transferase	None	None	c.G664-, 2 truncated proteins (263 and 251 aas)
lipopolysaccharide assembly protein LptD	Truncated version c.G1861-; p.*	None	None
*Efflux*			
BepE (efflux)	Shorter protein	Deletions in positions 370,371,381,1,641 (truncated protein, p.G125W, p.v126R, p.v127S)	Deletions in positions 370,371,381,1,641 (truncated protein, p.G125W, p.v126R, p.v127S)
BepG (efflux)	Deletion c.2867 (truncated protein)	Truncated version c.C1937T, Truncated protein	Deletion c.2867 (truncated protein)
Multidrug efflux transporter transcriptional repressor AcrR	Mutations p.K53R, p.K55N, p.K80N, p.N54I, p.N60T	Shorter version	Mutations p.K53R, p.K55N, p.K80N, p.N54I, p.N60T
Multidrug resistance protein MdtM	LGVLRDFRNVFRNRIF>WACCAIFATSFATASF	c.202 GILH>PRCRM	Lack 83 aa
*Secretion systems*			
T2SS protein F	Truncated version	Truncated version	Truncated version
T6SS VgrG1	c.G2182-, Truncated protein(p.N768*)	None	c.G2182-, Truncated protein (p.N768*)
*Fimbrial proteins*			
Fimbrial chaperone YadV	c.C624-; Truncated protein p.P210L	Truncated protein	Truncated protein
Putative hydrolase YxeP (active on CN bonds)	Truncated version	None	Truncated version
Outer membrane usher protein HtrE	Deletion 2,205 (truncated protein)	c.C2202-. Shorter sequence	Deletion 2,205 (truncated protein)
Xylose import ATP-binding protein XylG	Truncated version	Truncated version	Truncated version
*Putative receptors*			
Outer membrane channel OprM	Insertion in position 177 (1 truncated protein, lacking 18 aa)	c.G539-	Insertion in position 177 (1 truncated protein, lacking 18 aa)
Ail/Lom family outer membrane protein	Truncated version (65 aa)	None	Truncated version (65 aa)
Receptor vitamin B12 BtuB	Truncated version; truncated protein	None	Truncated version deletion 1,234, truncated protein p.S414P, p.L415S
Ferrichrome FhuA	None	None	2 truncated versions
Ferrienterobactin receptor (FepA)	3 truncated versions*	2 truncated versions	3 truncated versions*
*Virulence factors*			
Ail/Lom family outer membrane protein	Deletion c.193- (truncated protein)	None	Deletion c.193- (truncated protein)

“c.” corresponds to the nucleotide changes found in the coding sequences, with “-” representing a deletion, while “p.” stands for protein sequences, with asterisks representing a stop codon. The symbol “†” indicates that mutations are the same between the studied conditions.

Mutations in the core gene involved in Type VI secretion system, *vgrG*, were found in these same two strains but absent in the strain adapted only to mucin (K3674_ad15_M). Similarly, the same changes were found in the coding sequence of the outer membrane protein Ail (*attachment invasion locus*) of K3574_ad15_P and K3574_ad15_P + M, being intact in the isolate K3574_ad15_M. Interestingly, *fhuA*, the gene encoding a ferrichrome transporter protein, showed a frameshift mutation that led to two truncated versions of this protein in K3574_ad15_P + M, whereas this mutation was absent in K3574_ad15_P and K3574_ad15_M.

Mutations were found in genes involved in the metabolism of carbohydrates only for the strains adapted 15 days to mucin alone and to the phage in presence of mucin. For instance, nucleotide changes were observed in the gene *licC*, involved in the phosphoenolpyruvate-dependent sugar phosphotransferase system (PTS), in *rbsA*, *rbsB*, and *fruK*, encoding the ribose and fructose import ATP-binding proteins RbsA/RbsB and FruK, respectively, or in *malI*, encoding a maltose regulon regulatory protein. More nucleotide changes in other relevant genes are reported in Table 1.

## 4. Discussion

Most of the works studying the interactions between pathogenic bacteria and their phages are generally carried out in well-defined laboratory conditions. However, these microbes in a natural environment develop complex interactions on mucosal surfaces of the vertebrate host (de Freitas Almeida et al., 2022). In 2022, de Freitas et al. demonstrated that co-evolution of *Flavobacterium columnare* to its virulent phage V156 in presence of mucin dramatically increased the acquisition of spacers in the CRISPR arrays of *F. columnare*, thus increasing immunity to this phage (de Freitas Almeida et al., 2022). This work highlights the need to consider both biotic and abiotic variables if bacteriophages are to be used therapeutically. It is thus essential to take a close look at the study of how mucins and other mucosal components influence the acquisition of bacterial resistance towards lytic phages, so that the therapeutic potential of these could be better understood in the *in vivo* system.

Throughout the co-evolution, we observed that phage-resistant mutants arose at the first day post-infection (Figure 3), similar to what has been claimed in previous co-evolution works (Oechslin, 2018). Interestingly, both phage production (from 1 dpi onwards) and evolved *K. pneumoniae* populations seemed to stabilize over the days, consistently with other studies (Rendueles et al., 2023). This is likely due to the fact that vB_KpnS-VAC35 selects for resistant bacterial mutants. Due to its gelatinous nature, mucin limits the diffusion of bacteria through this space and facilitates the interaction of phages with bacteria, a less well-studied function but already documented in literature (Barr et al., 2013; de Freitas Almeida et al., 2022). In 2013, Barr et al. proposed a model in which bacteriophages would bind to mucins using the Ig-like domains present in many structural proteins, concentrate there, and protect humans and other metazoans against bacterial invaders (Barr et al., 2013; Almeida et al., 2019). Taken together, these two reasons could explain why the phage counts were higher in the presence of mucin than in the absence of this compound (Figure 3).

Interestingly, when the lytic phage was co-evolved to K3574 in the mucoid environment (vB_KpnS-VAC35_ad15_m), it showed an impaired infection ability compared to the adapted phage in the absence of mucin (vB_KpnS-VAC35_ad15) (Figure 4). As expected, no spot was visible for the cells exposed to the phage either in the presence or the absence of mucin, leading to the conclusion that resistance arose as a result of co-evolution. We phenotypically confirmed that mucin increased the frequency of resistant mutants for *K. pneumoniae* K3574 and K3325 strains (Figure 5). This resistant phenotype could be due to the modification of the phage receptor; however, as this strategy represents an important fitness cost for bacteria, these have developed other strategies to avoid phage attachment (Wright et al., 2019). For instance, receptors can be masked, preventing recognition while retaining function. Capsules or exopolysaccharides provide phage resistance in *Pseudomonas* spp. and *K. pneumoniae* (Hao et al., 2021), and these bacterial structures can be favorized in the presence of mucosal components such as mucins. Furthermore, NAC effectively reduced this frequency in the case of the cells incubated with this mucolytic (Figure 5). Numerous reports have claimed the absence of toxicity of NAC, an FDA-approved drug that has been in clinical practice for several decades, widely used in the case of acetaminophen intoxication (Bunchorntavakul and Reddy, 2018) and in several dermatologic conditions (such as trichotillomania, ichthyoses, dermatitis, melasma or pseudoporphyria) (Adil et al., 2018). NAC is a cheap, hydrophilic molecule, exhibiting an optimal toxicity profile (Mant et al., 1984; Mahmoudi et al., 2015). These properties make NAC an interesting compound for other therapeutic purposes, even a suitable adjuvant (De Flora et al., 2020; Biswas and Tk, 2022).

We wanted to have a better comprehension of the bacterial mutations in the co-evolution context, so we conducted an exhaustive genomic study (Figures 1, 6). The analysis of protein clusters suggested that the presence of the phage in this long-term co-evolution experiment was the main driver in the acquisition of mutations. The genomic analysis of *K. pneumoniae* K3574 adapted to the phage alone and in presence of mucin revealed mutations in some proteins involved in the bacterial defense to phages, such as methyltransferases, the HigA antitoxin, the *quorum sensing* autoinducer LsrB or the type 6 secretion system VgrG, as reported in other works (Liu et al., 2022; Blasco et al., 2023) (Figure 6). In the presence of mucin, mutations were observed in the genes encoding proteins that were involved in the carbohydrates metabolism, such as in the PTS system, which is a major carbohydrate active-transport system that catalyzes the phosphorylation of incoming sugar substrates concomitant with their translocation across the cell membrane (Deutscher et al., 2006).

Since changes concerning the synthesis, secretion or structure of mucins have been linked to gastrointestinal and respiratory disorders, manipulation of mucin may ultimately influence the microbiota and the effectiveness of phage therapy for bacterial imbalances (Carroll-Portillo and Lin, 2021), and the use of a mucolytic agent as an adjuvant of lytic phages could be an interesting therapeutic option to take into consideration. It has been shown that the presence of bacteria upregulates mucin production and enhances their encapsulation by mucin in the colon, so this could be even more important in CF patients in which overproduction of mucins leads to lung chronic infections (Bergstrom et al., 2020). Importantly, the trade-off costs that phage pressure and co-evolution represent for bacteria might render them less virulent in case of mutations in surface virulence factors, so maximizing the fitness costs that come with co-evolution may ultimately enhance the long-term efficacy of phage therapy. Optimization of these fitness costs could be a relevant factor to enhance the patient’s prognosis (Mangalea and Duerkop, 2020).

Among the limitations of this work, it is of highlight the fact that we performed an *in vitro* co-evolution between *K. pneumoniae* strains and a lytic bacteriophage, so the time of phage-resistance arising might be really different in the *in vivo* setting. Moreover, most of the infections in patients are indeed polymicrobial (Peters et al., 2012; Filkins and O’Toole, 2015). All in all, this study sheds some light in the phage resistance behavior that might be expected for some clinical strains of *K. pneumoniae* in a mucoid environment, and takes a deeper look at the rise in the phage-resistance that mucins impose to bacteria, already reported in literature (de Freitas Almeida et al., 2022). Evolutionary dynamics between bacterial pathogens and their natural predators in *in vivo* environments where mucin overproduction occurs deserve further investigation, which could help clinicians to predict the success of a particular phage administered to counteract infections. Finally, our results showed that an innovative option could be the application of mucolytic agents prior to the administration of lytic phages against *K. pneumoniae* infections, especially in environments where mucins are overproduced, as in cystic fibrosis disease. However, it would be necessary to carry out more studies that include a broader number of clinical isolates to confirm this innovative therapeutic option.

## Data availability statement

The datasets presented in this study can be found in online repositories. The names of the repository/repositories and accession number(s) can be found in the article/Supplementary Material.

## Author contributions

OP: Investigation, Methodology, Writing – original draft. LB: Investigation, Supervision, Writing – review & editing. CO: Investigation, Visualization, Writing – review & editing. IB: Investigation, Visualization, Writing – review & editing. LF-G: Writing – review & editing. ML: Investigation, Supervision, Visualization, Writing – review & editing. AB-P: Visualization, Writing – review & editing. FC: Visualization, Methodology, Writing – review & editing. BA: Visualization, Investigation, Writing – review & editing. JO-I: Visualization, Methodology, Writing – review & editing. MT: Writing – review & editing, Formal analysis, Funding acquisition, Investigation, Supervision, Validation.

## Footnotes

^1^ https://github.com/s-andrews/FastQC

^2^ https://github.com/wdecoster/NanoPlot

^3^ https://orthovenn2.bioinfotoolkits.net/home

## References

[ref1] Abedon S. T. Yin J. (2009). Bacteriophage plaques: theory and analysis. Methods Mol. Biol. 501, 161–174. doi: 10.1007/978-1-60327-164-6_17, PMID: 19066821

[ref2] Adil M. Amin S. S. Mohtashim M. (2018). N-acetylcysteine in dermatology. Indian J. Dermatol. Venereol. Leprol. 84, 652–659. doi: 10.4103/ijdvl.IJDVL_33_18, PMID: 30246706

[ref3] Almeida G. M. F. Laanto E. Ashrafi R. Sundberg L. R. (2019). Bacteriophage adherence to mucus mediates preventive protection against pathogenic bacteria. MBio 10:19. doi: 10.1128/mBio.01984-19, PMID: 31744913 PMC6867891

[ref4] Ambroa A. Blasco L. Lopez M. Pacios O. Bleriot I. Fernandez-Garcia L.. (2021). Genomic analysis of molecular bacterial mechanisms of resistance to phage infection. Front. Microbiol. 12:784949. doi: 10.3389/fmicb.2021.784949, PMID: 35250902 PMC8891609

[ref5] Barr J. J. Auro R. Furlan M. Whiteson K. L. Erb M. L. Pogliano J.. (2013). Bacteriophage adhering to mucus provide a non-host-derived immunity. Proc. Natl. Acad. Sci. U. S. A. 110, 10771–10776. doi: 10.1073/pnas.1305923110, PMID: 23690590 PMC3696810

[ref6] Bergstrom K. Shan X. Casero D. Batushansky A. Lagishetty V. Jacobs J. P.. (2020). Proximal colon-derived O-glycosylated mucus encapsulates and modulates the microbiota. Science 370, 467–472. doi: 10.1126/science.aay7367, PMID: 33093110 PMC8132455

[ref7] Biswas D. P. Tk D. S. (2022). The efficacy of adjuvant N acetyl cysteine for the eradication of H pylori infections: a systematic review and meta-analysis of randomized clinical trials. Clin. Res. Hepatol. Gastroenterol. 46:101832. doi: 10.1016/j.clinre.2021.101832, PMID: 34775122

[ref8] Blasco L. López-Hernández I. Rodríguez-Fernández M. Pérez-Florido J. Casimiro-Soriguer C. S. Djebara S.. (2023). Case report: analysis of phage therapy failure in a patient with a Pseudomonas aeruginosa prosthetic vascular graft infection. Front. Med. 10:1199657. doi: 10.3389/fmed.2023.1199657, PMID: 37275366 PMC10235614

[ref9] Bleriot I. Blasco L. Pacios O. Fernández-García L. López M. Ortiz-Cartagena C.. (2023). Proteomic study of the interactions between phages and the bacterial host. Microbiol Spectr 11:e0397422. doi: 10.1128/spectrum.03974-22, PMID: 36877024 PMC10100988

[ref10] Bunchorntavakul C. Reddy K. R. (2018). Acetaminophen (APAP or N-acetyl-p-aminophenol) and acute liver failure. Clin. Liver Dis. 22, 325–346. doi: 10.1016/j.cld.2018.01.007, PMID: 29605069

[ref11] Carroll-Portillo A. Lin H. C. (2021). Exploring mucin as adjunct to phage therapy. Microorganisms 9:509. doi: 10.3390/microorganisms9030509, PMID: 33670927 PMC7997181

[ref12] Castillo J. A. Secaira-Morocho H. Maldonado S. Sarmiento K. N. (2020). Diversity and evolutionary dynamics of Antiphage defense Systems in Ralstonia solanacearum species complex. Front. Microbiol. 11:961. doi: 10.3389/fmicb.2020.00961, PMID: 32508782 PMC7251935

[ref13] De Flora S. Balansky R. La Maestra S. (2020). Rationale for the use of N-acetylcysteine in both prevention and adjuvant therapy of COVID-19. FASEB J. 34, 13185–13193. doi: 10.1096/fj.202001807, PMID: 32780893 PMC7436914

[ref14] de Freitas Almeida G. M. Hoikkala V. Ravantti J. Rantanen N. Sundberg L. R. (2022). Mucin induces CRISPR-Cas defense in an opportunistic pathogen. Nat. Commun. 13:3653. doi: 10.1038/s41467-022-31330-3, PMID: 35752617 PMC9233685

[ref15] Dedrick R. M. Guerrero-Bustamante C. A. Garlena R. A. Russell D. A. Ford K. Harris K.. (2019). Engineered bacteriophages for treatment of a patient with a disseminated drug-resistant Mycobacterium abscessus. Nat. Med. 25, 730–733. doi: 10.1038/s41591-019-0437-z, PMID: 31068712 PMC6557439

[ref16] Delfino E. Giacobbe D. R. Del Bono V. Coppo E. Marchese A. Manno G.. (2015). First report of chronic pulmonary infection by KPC-3-producing and colistin-resistant Klebsiella pneumoniae sequence type 258 (ST258) in an adult patient with cystic fibrosis. J. Clin. Microbiol. 53, 1442–1444. doi: 10.1128/JCM.03199-14, PMID: 25653395 PMC4365242

[ref17] Denes T. den Bakker H. C. Tokman J. I. Guldimann C. Wiedmann M. (2015). Selection and characterization of phage-resistant mutant strains of Listeria monocytogenes reveal host genes linked to phage adsorption. Appl. Environ. Microbiol. 81, 4295–4305. doi: 10.1128/AEM.00087-15, PMID: 25888172 PMC4475870

[ref18] Deutscher J. Francke C. Postma P. W. (2006). How phosphotransferase system-related protein phosphorylation regulates carbohydrate metabolism in bacteria. Microbiol. Mol. Biol. Rev. 70, 939–1031. doi: 10.1128/MMBR.00024-06, PMID: 17158705 PMC1698508

[ref19] Filkins L. M. O’Toole G. A. (2015). Cystic fibrosis lung infections: polymicrobial, complex, and hard to treat. PLoS Pathog. 11:e1005258. doi: 10.1371/journal.ppat.1005258, PMID: 26719892 PMC4700991

[ref20] Green S. I. Liu C. G. Yu X. Gibson S. Salmen W. Rajan A.. (2021). Targeting of mammalian Glycans enhances phage predation in the gastrointestinal tract. MBio 12:20. doi: 10.1128/mBio.03474-20, PMID: 33563833 PMC7885116

[ref21] Hansson G. C. (2019). Mucus and mucins in diseases of the intestinal and respiratory tracts. J. Intern. Med. 285, 479–490. doi: 10.1111/joim.12910, PMID: 30963635 PMC6497544

[ref22] Hao G. Shu R. Ding L. Chen X. Miao Y. Wu J.. (2021). Bacteriophage SRD2021 recognizing capsular polysaccharide shows therapeutic potential in serotype K47 Klebsiella pneumoniae infections. Antibiotics 10:894. doi: 10.3390/antibiotics10080894, PMID: 34438943 PMC8388747

[ref23] Jass J. R. Walsh M. D. (2001). Altered mucin expression in the gastrointestinal tract: a review. J. Cell. Mol. Med. 5, 327–351. doi: 10.1111/j.1582-4934.2001.tb00169.x, PMID: 12067494 PMC6517815

[ref24] Kamruzzaman M. Iredell J. R. (2019). CRISPR-Cas system in antibiotic resistance plasmids in Klebsiella pneumoniae. Front. Microbiol. 10:2934. doi: 10.3389/fmicb.2019.02934, PMID: 31998256 PMC6965323

[ref25] Kutter E. (2009). Phage host range and efficiency of plating. Methods Mol. Biol. 501, 141–149. doi: 10.1007/978-1-60327-164-6_14 19066818

[ref26] Law N. Logan C. Yung G. Furr C. L. Lehman S. M. Morales S.. (2019). Successful adjunctive use of bacteriophage therapy for treatment of multidrug-resistant Pseudomonas aeruginosa infection in a cystic fibrosis patient. Infection 47, 665–668. doi: 10.1007/s15010-019-01319-0, PMID: 31102236

[ref27] Leão R. S. Pereira R. H. Folescu T. W. Albano R. M. Santos E. A. Junior L. G.. (2011). KPC-2 carbapenemase-producing Klebsiella pneumoniae isolates from patients with cystic fibrosis. J. Cyst. Fibros. 10, 140–142. doi: 10.1016/j.jcf.2010.12.003, PMID: 21220216

[ref28] Liu M. Hernandez-Morales A. Clark J. Le T. Biswas B. Bishop-Lilly K. A.. (2022). Comparative genomics of Acinetobacter baumannii and therapeutic bacteriophages from a patient undergoing phage therapy. Nat. Commun. 13:3776. doi: 10.1038/s41467-022-31455-5, PMID: 35773283 PMC9247103

[ref29] Lopatina A. Tal N. Sorek R. (2020). Abortive infection: bacterial suicide as an antiviral immune strategy. Annu Rev Virol 7, 371–384. doi: 10.1146/annurev-virology-011620-040628, PMID: 32559405

[ref30] Lopes A. Pereira C. Almeida A. (2018). Sequential combined effect of phages and antibiotics on the inactivation of Escherichia coli. Microorganisms 6:125. doi: 10.3390/microorganisms6040125, PMID: 30563133 PMC6313441

[ref31] Mackow N. A. Shen J. Adnan M. Khan A. S. Fries B. C. Diago-Navarro E. (2019). CRISPR-Cas influences the acquisition of antibiotic resistance in Klebsiella pneumoniae. PLoS One 14:e0225131. doi: 10.1371/journal.pone.0225131, PMID: 31747398 PMC6867608

[ref32] Mahmoudi G. A. Astaraki P. Mohtashami A. Z. Ahadi M. (2015). N-acetylcysteine overdose after acetaminophen poisoning. Int Med Case Rep J 8, 65–69. doi: 10.2147/IMCRJ.S74563, PMID: 25767408 PMC4354467

[ref33] Majkowska-Skrobek G. Markwitz P. Sosnowska E. Lood C. Lavigne R. Drulis-Kawa Z. (2021). The evolutionary trade-offs in phage-resistant Klebsiella pneumoniae entail cross-phage sensitization and loss of multidrug resistance. Environ. Microbiol. 23, 7723–7740. doi: 10.1111/1462-2920.15476, PMID: 33754440

[ref34] Mangalea M. R. Duerkop B. A. (2020). Fitness trade-offs resulting from bacteriophage resistance potentiate synergistic antibacterial strategies. Infect. Immun. 88:19. doi: 10.1128/IAI.00926-19, PMID: 32094257 PMC7309606

[ref35] Mant T. G. Tempowski J. H. Volans G. N. Talbot J. C. (1984). Adverse reactions to acetylcysteine and effects of overdose. Br. Med. J. (Clin. Res. Ed.) 289, 217–219. doi: 10.1136/bmj.289.6439.217, PMID: 6234965 PMC1442311

[ref36] Moulton-Brown C. E. Friman V. P. (2018). Rapid evolution of generalized resistance mechanisms can constrain the efficacy of phage-antibiotic treatments. Evol. Appl. 11, 1630–1641. doi: 10.1111/eva.12653, PMID: 30344632 PMC6183449

[ref37] Oechslin F. (2018). Resistance development to bacteriophages occurring during bacteriophage therapy. Viruses 10:351. doi: 10.3390/v10070351, PMID: 29966329 PMC6070868

[ref39] Pacios O. Blasco L. Bleriot I. Fernandez-Garcia L. Gonzalez Bardanca M. Ambroa A.. (2020). Strategies to combat multidrug-resistant and persistent infectious diseases. Antibiotics 9:65. doi: 10.3390/antibiotics9020065, PMID: 32041137 PMC7168131

[ref40] Pacios O. Fernández-García L. Bleriot I. Blasco L. González-Bardanca M. López M.. (2021). Enhanced antibacterial activity of repurposed Mitomycin C and Imipenem in combination with the lytic phage vB_KpnM-VAC13 against clinical isolates of Klebsiella pneumoniae. Antimicrob. Agents Chemother. 65:e0090021. doi: 10.1128/AAC.00900-21, PMID: 34228538 PMC8370222

[ref41] Paone P. Cani P. D. (2020). Mucus barrier, mucins and gut microbiota: the expected slimy partners? Gut 69, 2232–2243. doi: 10.1136/gutjnl-2020-322260, PMID: 32917747 PMC7677487

[ref42] Peters B. M. Jabra-Rizk M. A. O’May G. A. Costerton J. W. Shirtliff M. E. (2012). Polymicrobial interactions: impact on pathogenesis and human disease. Clin. Microbiol. Rev. 25, 193–213. doi: 10.1128/CMR.00013-11, PMID: 22232376 PMC3255964

[ref43] Pletzer D. Mansour S. C. Wuerth K. Rahanjam N. Hancock R. E. (2017). New mouse model for chronic infections by gram-negative bacteria enabling the study of anti-infective efficacy and host-microbe interactions. MBio 8:17. doi: 10.1128/mBio.00140-17, PMID: 28246361 PMC5347345

[ref44] Raya R. R. H’bert E. M. (2009). Isolation of phage via induction of Lysogens. Methods Mol. Biol. 501, 23–32. doi: 10.1007/978-1-60327-164-6_3, PMID: 19066807

[ref45] Rendueles O. de Sousa J. A. M. Rocha E. P. C. (2023). Competition between lysogenic and sensitive bacteria is determined by the fitness costs of the different emerging phage-resistance strategies. elife 12:83479. doi: 10.7554/eLife.83479, PMID: 36975200 PMC10076033

[ref46] Riquelme S. A. Ahn D. Prince A. (2018). Pseudomonas aeruginosa and Klebsiella pneumoniae adaptation to innate immune clearance mechanisms in the lung. J. Innate Immun. 10, 442–454. doi: 10.1159/000487515, PMID: 29617698 PMC6785651

[ref47] Schooley R. T. Biswas B. Gill J. J. Hernandez-Morales A. Lancaster J. Lessor L.. (2017). Development and use of personalized bacteriophage-based therapeutic cocktails to treat a patient with a disseminated resistant Acinetobacter baumannii infection. Antimicrob. Agents Chemother. 61:17. doi: 10.1128/AAC.00954-17, PMID: 28807909 PMC5610518

[ref48] Seemann T. (2014). Prokka: rapid prokaryotic genome annotation. Bioinformatics 30, 2068–2069. doi: 10.1093/bioinformatics/btu153, PMID: 24642063

[ref49] Uyttebroek S. Chen B. Onsea J. Ruythooren F. Debaveye Y. Devolder D.. (2022). Safety and efficacy of phage therapy in difficult-to-treat infections: a systematic review. Lancet Infect. Dis. 22, e208–e220. doi: 10.1016/S1473-3099(21)00612-5, PMID: 35248167

[ref50] Wright R. C. T. Friman V. P. Smith M. C. M. Brockhurst M. A. (2019). Resistance evolution against phage combinations depends on the timing and order of exposure. MBio 10:19. doi: 10.1128/mBio.01652-19, PMID: 31551330 PMC6759759

[ref51] Xu Q. Yang X. Chan E. W. C. Chen S. (2021). The hypermucoviscosity of hypervirulent K. pneumoniae confers the ability to evade neutrophil-mediated phagocytosis. Virulence 12, 2050–2059. doi: 10.1080/21505594.2021.1960101, PMID: 34339346 PMC8331041

