# Supplementary material for: Molecular studies of phages-Klebsiella pneumoniae in mucoid environment: innovative use of mucolytic agents prior to the administration of lytic phages
Source: Front Microbiol. 2023 Oct 11;14:1286046. doi: 10.3389/fmicb.2023.1286046 (PMC10598653; doi:10.3389/fmicb.2023.1286046)
Supplement: Supplementary file 1 [file Data_Sheet_1.PDF]

Supplementary material:

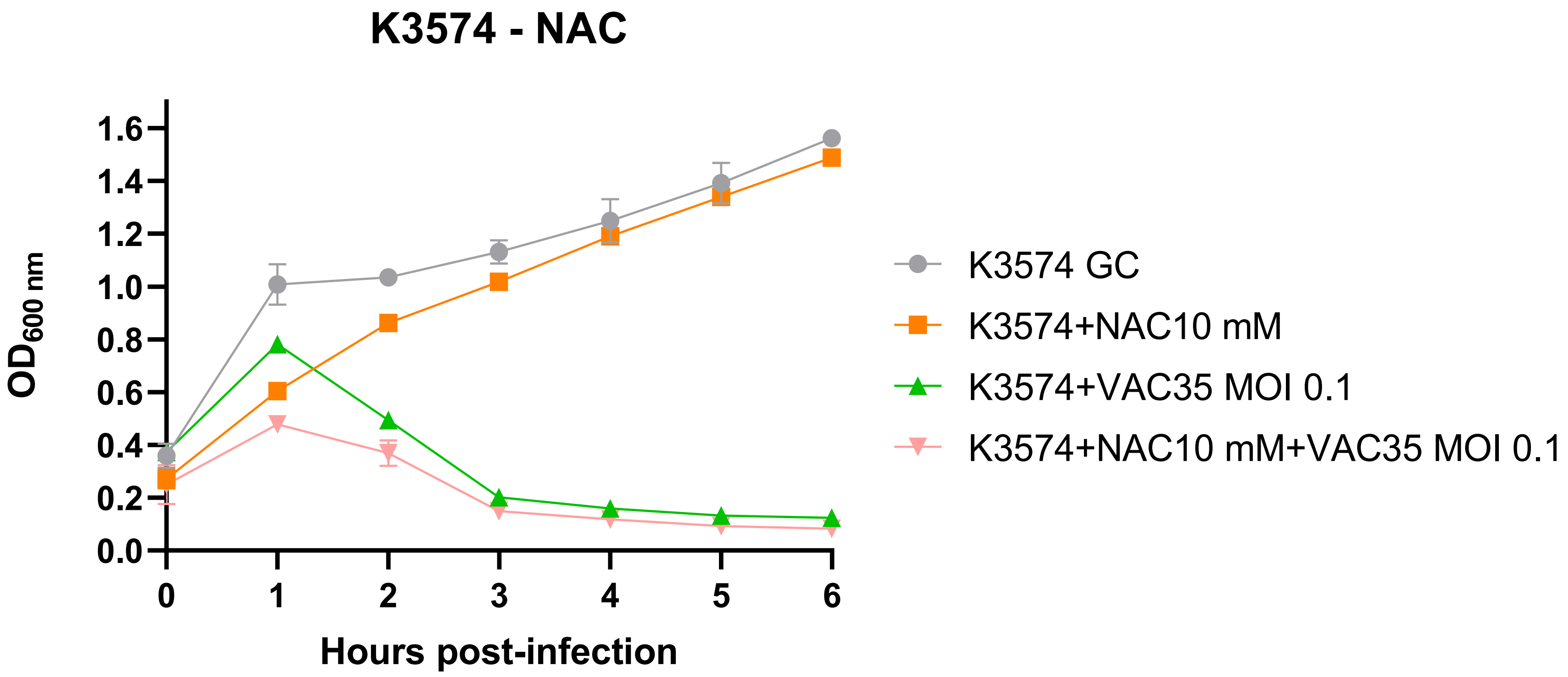

Growth curve in presence of NAC (10 mM) and the lytic phage vB_KpnS_VAC35 at MOI 0.1 to demonstrate the absence of interaction between both agents on the clinical isolate of *K. pneumoniae* K3574
